# Mapping and characterization of rust resistance genes *Lr53* and *Yr35* introgressed from *Aegilops* species

**DOI:** 10.1007/s00122-024-04616-x

**Published:** 2024-04-28

**Authors:** Binyang Xu, Tao Shen, Hong Chen, Hongna Li, Shams ur Rehman, Shikai Lyu, Lei Hua, Guiping Wang, Chaozhong Zhang, Kairong Li, Hao Li, Caixia Lan, Guo-Yue Chen, Ming Hao, Shisheng Chen

**Affiliations:** 1 National Key Laboratory of Wheat Improvement, Peking University Institute of Advanced Agricultural Sciences, Shandong Laboratory of Advanced Agriculture Sciences in Weifang, Weifang, 261325 Shandong China; 2 Triticeae Research Institute, Sichuan Agricultural University, Chengdu, 611130 Sichuan China; 3 Institute of Genetics and Developmental Biology, Chinese Academy of Sciences, Beijing, 100000 China; 4 Department of Plant Sciences, University of California, Davis, CA 95616 USA; 5 State Key Laboratory of Crop Stress Adaptation and Improvement, College of Agriculture, Henan University, Kaifeng, 475004 China; 6 National Key Laboratory of Crop Genetic Improvement, Huazhong Agricultural University, Wuhan, 430070 China

## Abstract

**Key message:**

The rust resistance genes* Lr53* and* Yr35* were introgressed into bread wheat from* Aegilops longissima *or* Aegilops sharonensis *or their S-genome containing species and mapped to the telomeric region of chromosome arm 6BS.

**Abstract:**

Wheat leaf and stripe rusts are damaging fungal diseases of wheat worldwide. Breeding for resistance is a sustainable approach to control these two foliar diseases. In this study, we used SNP analysis, sequence comparisons, and cytogenetic assays to determine that the chromosomal segment carrying *Lr53* and *Yr35* was originated from *Ae.longissima* or *Ae. sharonensis* or their derived species. In seedling tests, *Lr53* conferred strong resistance against all five Chinese *Pt* races tested, and *Yr35* showed effectiveness against *Pst* race CYR34 but susceptibility to race CYR32. Using a large population (3892 recombinant gametes) derived from plants homozygous for the *ph1b* mutation obtained from the cross 98M71 × CS*ph1b*, both *Lr53* and *Yr35* were successfully mapped to a 6.03-Mb telomeric region of chromosome arm 6BS in the Chinese Spring reference genome v1.1. Co-segregation between *Lr53* and *Yr35* was observed within this large mapping population. Within the candidate region, several nucleotide-binding leucine-rich repeat genes and protein kinases were identified as candidate genes. Marker *pku6B3127* was completely linked to both genes and accurately predicted the absence or presence of alien segment harboring *Lr53* and *Yr35* in 87 tetraploid and 149 hexaploid wheat genotypes tested. We developed a line with a smaller alien segment (< 6.03 Mb) to reduce any potential linkage drag and demonstrated that it conferred resistance levels similar to those of the original donor parent 98M71. The newly developed introgression line and closely linked PCR markers will accelerate the deployment of *Lr53* and *Yr35* in wheat breeding programs.

**Supplementary Information:**

The online version contains supplementary material available at 10.1007/s00122-024-04616-x.

## Introduction

Bread wheat (*Triticum aestivum* L., 2*n* = 6*x* = 42, AABBDD) is one of the most important cereal crops providing approximately 20% of the food calories and protein for more than 4.5 billion people (Gupta et al. 2008). Reducing yield losses caused by fungal diseases is an effective way to enhance wheat production. *Puccinia triticina* Eriksson (*Pt*) and *Puccinia striiformis* f. sp. *tritici* (*Pst*), the causal agents of wheat leaf rust and stripe rust, respectively, are two devastating fungal diseases threatening global wheat production. In recent years, *Pt* and *Pst* pathogens have become increasingly problematic due to the emergence of widely virulent races (Boshoff et al. 2018; Han et al. 2015; Hovmøller et al. 2015; Milus et al. 2009; Omara et al. 2021).

New *Pst* races virulent on wheat genotypes with *Yr5* have been reported in various countries, including China, Australia, India, and Turkey (Tekin et al. 2021; Zhang et al. 2022). Race CYR32 was responsible for severe stripe rust epidemics in China in 2001/2002, which led to significant yield losses across approximately 6.6 million hectares of wheat area (Wan et al. 2004). This strain continues to be one of the most predominant races in China even today (Wang et al. 2018). Race CYR34 was first isolated in Sichuan Province, China, in 2008 and was virulent to *Yr24*/*Yr26* and *Yr10*, resulting in many bread wheat cultivars carrying these *Yr* genes becoming susceptible (Liu et al. 2010; Wang et al. 2019). The *Pt* races THTT, THTS, THJT, THJS, PHTT, and PHJT were the most common races in China and showed virulence to many *Lr* genes, including *Lr1*, *Lr2a*, *Lr2b*, *Lr2c*, *Lr3*, *Lr3bg*, *Lr10*, *Lr11*, *Lr14a*, *Lr14b*, *Lr16*, *Lr17*, *Lr26*, *Lr32*, *LrB*, *Lr33*, and *Lr50* (Zhang et al. 2020). China encountered severe leaf rust epidemics in the years 2012, 2013, and 2015, leading to significant reductions in yield (Zhang et al. 2020). Although fungicides are available for controlling these rust diseases, they are expensive and may pose risks to human health and the environment. Hence, more *Pt* and *Pst* resistance genes are needed to diversify the combinations of deployed resistance genes to minimize the risks associated with relying on limited sources of resistance.

So far, approximately 83 leaf rust resistance (*Lr*) genes and 86 stripe rust resistance (*Yr*) genes have been cataloged in wheat and its wild relatives (Kolmer et al. 2023; Zhu et al. 2023). Among these, all-stage resistance (ASR) and adult-plant resistance (APR) genes are the two major types of rust resistance genes (Chen 2005). Most of these *Lr* and *Yr* genes are ASR genes, which exhibit efficacy in both seedling and adult-plant stages. However, owing to the size and complexity of wheat genomes, only 11 *Lr* genes (*Lr1*, *Lr9*/*Lr58*, *Lr10*, *Lr13*, *Lr14a*, *Lr21*, *Lr22a*, *Lr34*, *Lr42*, *Lr47*, and *Lr67*) and ten *Yr* genes (*Yr5*/*YrSP*, *Yr7*, *Yr27*, *Yr15*, *Yr18*, *Yr36*, *Yr46*, *Yr28*, *YrU1*, and *Yr10*/*YrNAM*) have been cloned to date (Li et al. 2023; Ni et al. 2023) either by map-based cloning or by rapid gene-cloning methods, including MutChromSeq, MutRenSeq, MutIsoSeq, and STAM (Ni et al. 2023; Sánchez-Martín et al. 2016; Steuernagel et al. 2016; Wang et al. 2023b). Among the cloned *Lr* and *Yr* genes, *Lr34*/*Yr18*/*Sr57*/*Pm38*, *Lr67*/*Yr46*/*Sr55*/*Pm46*, and *Yr36* are APR genes encoding a putative ATP-binding cassette transporter, a hexose transporter, and a kinase-START protein, respectively (Krattinger et al. 2009; Moore et al. 2015; Fu et al. 2009).

Wild relatives of wheat have previously been utilized for transferring *Lr* and *Yr* genes into common wheat varieties, including *Yr34*, *QYrtm.pau-2A*, *QYrtb.pau-5A*, *LrPI119435*, and *Lr63* from *T. monococcum* (Chen et al. 2021; Chhuneja et al. 2008; Kolmer et al. 2010; Wang et al. 2023a); *Yr15*, *Yr36*, and *Lr64* from *T. dicoccoides* (Klymiuk et al. 2018; Ren et al. 2023); *Lr21*, *Lr22a*, *Lr32*, *Lr39*-*Lr43*, and *Yr28* from *Ae. tauschii* (Athiyannan et al. 2022b; Ren et al. 2023); *Lr25*, *Lr26*, *Lr45*, *Yr9*, and *Yr83* from *Secale cereale* (Li et al. 2020; Spetsov and Daskalova 2022); *Lr28*, *Lr35*, *Lr36*, *Lr47*, *Lr51*, and *Lr66* from *Ae. speltoides* (Li et al. 2023; Marais et al. 2010b); *Lr62* and *Yr42* from *Ae. neglecta* (Marais et al. 2009); and *Lr56* and *Yr38* from *Ae. sharonensis* (Marais et al. 2010a). A recent study showed that the leaf and stripe rust resistance gene *Lr/Yr548* was originated from *Ae. sharonensis* and *Ae. longissima*, which are closely related diploid species of the section *Sitopsis* (Sharon et al. 2023).

The two linked all-stage resistance genes *Lr53* and *Yr35* were introduced from *T. dicoccoides* accession 479 into hexaploid wheat and were mapped on chromosome arm 6BS using monosomic analyses and telocentric mapping (Marais et al. 2003, 2005a). *Lr53* confers high resistance to at least 55 individual *Pt* races and five inoculum mixtures of *Pt* from North America, South Africa, India, and Australia, and *Yr35* exhibits effectiveness against 11 *Pst* races from the same regions (Dadkhodaie et al. 2011; Dong et al. 2017; Marais et al. 2018, 2005a; Raghunandan et al. 2022). Leaf and stripe rust isolates virulent on *Lr53* and *Yr35* have not yet been identified, making them potentially useful for breeding rust-resistant wheat cultivars. The objectives of this study were to: (1) test whether *Lr53* and *Yr35* confer resistance against *Pt* and *Pst* races prevalent in China, (2) verify the origin of the introgressed chromosomal segment carrying these two genes, and (3) generate precise genetic maps and identify potential candidate genes associated with *Lr53* and *Yr35*.

## Materials and methods

### Plant materials and mapping populations

As a source of the rust resistance genes *Lr53* and *Yr35*, we used wheat accessions 98M71 (PI 648417; pedigree: *T. dicoccoides*-479/4*CS//3*CS-S/3/CS) and Thatcher-*Lr53* (PI 682091; pedigree: CS*4/*T. dicoccoides*-479//3*CS-S/3/CS/4/5*Thatcher). These accessions are near-isogenic lines to wheat varieties Chinese spring (CS) and Thatcher, respectively (Marais et al. 2018, 2005a). The introgression line 98M71 was crossed with the susceptible wheat line Avocet-S and the CS *ph1b* mutant (CS*ph1b*) to generate two mapping populations. PCR Markers *Xwgc2049* and *Xwgc2111* were used to confirm the absence of the *Ph1* gene (Gyawali et al. 2019). We evaluated a subset of 136 F_2_ plants from the 98M71 × Avocet-S cross using *Pt* race PHQS, and another subset of 117 F_2_ plants from the same population with *Pst* race CYR34. The second population (98M71 × CS*ph1b*), which included 1,946 plants derived from selected F_3_ families that were homozygous for the *ph1b* mutation and segregating for the introgressed alien segment carrying *Lr53* and *Yr35*, was used to construct the genetic linkage maps. Eight *Ae. longissima* and two *Ae. sharonensis* accessions obtained from the Chinese Crop Germplasm Resources Information System (https://www.cgris.net/) were evaluated using PCR markers derived from the introgressed segment of 98M71. Finally, we used a collection of 87 accessions of *T. turgidum* (including *T. dicoccon*, *T. dicoccoides*, and *T. durum*) and 149 accessions of *T. aestivum* to determine the value of the tightly linked PCR markers identified in the present study for marker-assisted selection.

### Leaf rust and stripe rust assays

The leaf rust and stripe rust seedling assays for both the parental lines and the mapping populations were conducted at the Peking University Institute of Advanced Agricultural Sciences, Weifang, China. The avirulence/virulence profiles of the *Pt* races (PHQS, THDB, PHRT, PHTT, and FHJR) and the *Pst* races (CYR32 and CYR34) used in this study can be found in Supplementary Table 1. Seedlings at the three-leaf stage were subjected to challenge with fresh urediniospores of *Pt* or *Pst* (1:30 talcum powder) using the shaking off method (Chen et al. 2021). The inoculation, incubation, and scoring of disease responses followed established procedures detailed in previous studies (Chen et al. 2021; Stakman et al. 1962). For plants carrying recombination events within the candidate region, we performed progeny tests including approximately 25 plants from each F_3:4_ family inoculated with *Pt* or *Pst* races. The infection types (ITs) of wheat plants were then scored using a 0–4 scale (Chen et al. 2021).

### Sequencing and bioinformatics analysis

RNA-seq of 98M71 was carried out at Novogene Bioinformatics Technology Co., Ltd. (Beijing, China). The raw sequencing data have been deposited at the National Genomics Data Center (NGDC) under the BioProject accession number PRJCA022411. Exome-capture data for the hexaploid wheat accession Avocet-S were downloaded from the T3/Wheat database (https://triticeaetoolbox.org/wheat/). The published reference genomes of *T. dicoccoides* (Zavitan) (Avni et al. 2017), *T. durum* (Svevo) (Maccaferri et al. 2019), *T. urartu* (G1812) (Ling et al. 2018), *T. monococcum* (PI 306540) (Wang et al. 2023a), *Aegilops tauschii* (AL8/78) (Luo et al. 2017), five *Sitopsis* species of *Aegilops* (TS01, TE01, TB01, TH02, TL05, AS_1644, and AEG-6782-2) (Avni et al. 2022; Li et al. 2022), and *T. aestivum* (CS, Arina*LrFor*, Attraktion, Fielder, Jagger, Julius, Kariega, Kenong 9204, LongReach Lancer, CDC Landmark, Mace, Norin61, Renan, CDC Stanley, and SY Mattis) were used for comparative analysis (Athiyannan et al. 2022a; Sato et al. 2021; Shi et al. 2022; The International Wheat Genome Sequencing Consortium 2018; Walkowiak et al. 2020). Raw reads of 98M71 were quality-trimmed using Trimmomatic v0.32 (Bolger et al. 2014). The trimmed reads were then aligned to the reference genome of CS using STAR v2.7.10a (Dobin et al. 2013). Freebayes v1.3.6 and BCFtools v1.14 were used for variant calling and filtering (Garrison and Marth 2012). Single-nucleotide polymorphisms (SNPs) were utilized to determine the size of the alien chromosome segment introgressed into bread wheat. Sequences were aligned using Muscle as implemented in software Mega v7.0 (Kumar et al. 2016). A phylogenetic tree was generated using the neighbor-joining method, and the resulting tree was visualized using Interactive Tree Of Life (iTOL) v5.0 (https://itol.embl.de/).

### Development of PCR markers

To amplify gene regions harboring putative polymorphisms, genome-specific primer pairs were designed using the Primer3 software (https://bioinfo.ut.ee/primer3-0.4.0/primer3/). The identified polymorphic sites were then utilized for developing two types of markers: cleaved amplified polymorphic sequence (CAPS) and insertion–deletion (InDel) markers (Bhattramakki et al. 2002; Konieczny and Ausubel 1993). PCR reactions were carried out in a Veriti 96-Well Fast Thermal Cycler (Applied Biosystems, USA). The PCR products that exhibited the expected sizes were subjected to Sanger sequencing to verify the presence of the expected polymorphisms. Restriction enzymes for digestion of the PCR products were purchased from New England BioLabs Inc. (Hitchin, UK) and employed according to established protocols.

### qRT-PCR analysis

At the three-leaf stage, 98M71 plants were inoculated with *Pt* (race THDB mixed with talcum powder) or with mock (talcum powder) in two independent growth chambers under identical environmental conditions, including a temperature regime of 24 °C during the day and 22 °C at night with a photoperiod of 16 h light and 8 h dark. Meanwhile, 98M71 plants were inoculated with *Pst* (race CYR34 mixed with talcum powder) or with mock (talcum powder) in another two independent growth chambers set at 18 °C during the day and 15 °C during the night. The *Pt-*/*Pst*-inoculated leaves and the mock-inoculated leaves from different plants were collected at 6 days post inoculation (dpi) and immediately stored in liquid nitrogen. Total RNA extraction was performed using the spectrum plant total RNA kit (MilliporeSigma, MA, USA). RNA-seq was carried out at Novogene Bioinformatics Technology Co., Ltd. (Beijing, China). Differentially expressed genes (DEGs) between *Pt-*/*Pst*-inoculated and mock-treated samples were identified using the edgeR software, employing significance thresholds of FDR < 0.05, *p*-value < 0.05, and |log2foldchange|> 1 (Robinson et al. 2010). Furthermore, qRT-PCR validation of candidate DEGs was performed using an ABI QuantStudio 5 real-time PCR system (Applied Biosystems, CA, USA). The transcript levels were determined in four biological replicates and quantified as fold-*ACTIN* levels (Chen et al. 2018; Zhang et al. 2017).

### Cytogenetic assays

For genomic in situ hybridization (GISH), the genomic DNA of *Ae. longissima* (S^l^S^l^) and *Ae. sharonensis* (S^sh^S^sh^) was labeled using the Atto550 NT labeling kit and Atto488 NT labeling kit (Jena Bioscience, Jena, Germany), respectively. The genomic DNA of CS was used as blocking DNA. For fluorescence in situ hybridization (FISH), the probes Oligo-pSc119.2 (Tang et al. 2014), Oligo-pTa535 (Tang et al. 2014), and Oligo-pTa-713 (Zhao et al. 2016) were employed for the identification of chromosomes of common wheat and *Aegilops* species. These synthetic oligonucleotides were labeled at the 5′ end with 6-carboxyfluorescein (6-FAM) or 6-carboxytetramethylrhodamine (Tamra) by Sangon Biotech Co. (Shanghai, China). The in situ hybridization procedure was conducted using the methods described previously (Fan et al. 2023). Chromosome preparations were counterstained with DAPI (4′,6-diamidino2-phenylindole) in Vectashield (Vector Laboratories, Burlingame, USA). Images were captured using a BX-63 microscope (Olympus, Japan) equipped with a Photometric SenSys Olympus DP70 CCD camera.

### Transferring of the truncated alien segment carrying *Lr53* and *Yr35* to hexaploid wheat

The recombinant obtained from the 98M71 × CS*ph1b* mapping population, possessing the truncated alien segment but carrying *Lr53* and *Yr35*, was subjected to crosses and backcrosses with the Chinese bread wheat cultivar Yangmai21 (YM21). YM21 is known to be susceptible to many *Pt* races, including THDB, PHRT, PHTT, PHQS, FHJL, and HCJR (Li et al. 2023). Flanking and completely linked DNA markers were used to validate the presence of the truncated alien segment in each generation. BC_1_F_1_ plants heterozygous for the truncated alien segment were self-pollinated. Subsequently, selected BC_1_F_2_ plants homozygous for the 98M71 allele were divided into six groups and grown in six independent growth chambers. Each group was inoculated with specific rust races: *Pst* race CYR34 or *Pt* races THDB, PHQS, PHTT, PHRT, and FHJR.

### Statistical analyses

The polymorphic PCR markers and the rust resistance phenotypes were used to construct the genetic maps of *Lr53* and *Yr35* using the software MapChart v2.2 (https://www.wur.nl/en/show/Mapchart.htm) (Voorrips 2002). The significance of the differences in transcript levels was estimated using two-sided unpaired *t*-test.

## Results

### Characterization of leaf rust and stripe rust resistance in wheat line 98M71

**Fig. 1:**
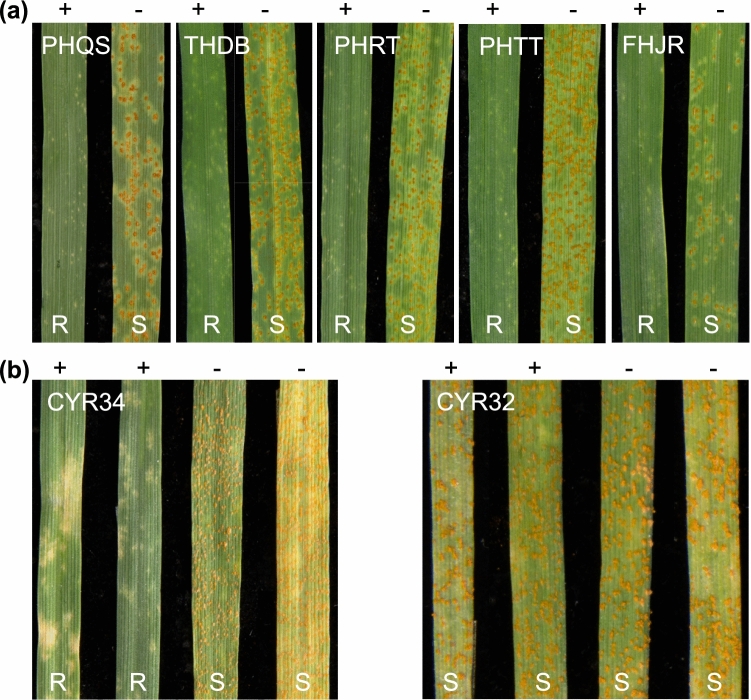
Infection types of 98M71 and its recurrent parent CS in response to *Pt* and *Pst* races. **a** Susceptibility or resistance responses of 98M71 and CS to *Pt* races PHQS, THDB, PHRT, PHTT, and FHJR. **b** Susceptibility or resistance responses of 98M71 and CS to *Pst* races CYR32 and CYR34. The presence or absence of the resistant allele is indicated by + or −, respectively. R, resistant; S, susceptible

Seedling tests revealed that the introgression line 98M71 exhibited strong resistance (ITs = 0; to 1) against all five *Pt* races tested, while its recurrent parent CS showed susceptibility with ITs ranging from 3+ to 4 (Fig. 1a). When evaluated against Chinese *Pst* races CYR32 and CYR34, 98M71 displayed high resistance (ITs = 0; to ;1-) against *Pst* race CYR34, but was susceptible (ITs = 3+) to the other race CYR32. By contrast, the recurrent parent CS exhibited susceptible infection types (ITs = 3+) to both *Pst* races (Fig. 1b).

In a subset of 136 F_2_ plants from the 98M71 × Avocet-S cross inoculated with *Pt* race PHQS, 91 plants were resistant (ITs = 0; to ;1-) and 45 were classified as susceptible (ITs = 3+ to 4). This observed ratio deviated slightly from the expected ratio of 3:1 (resistant : susceptible), with an excess of susceptible plants (*χ*^*2*^ = 4.74, *p* = 0.029). In another subset consisting of 117 F_2_ individuals from the same population inoculated with *Pst* race CYR34, 79 plants exhibited resistance (ITs = 0; to 1) and 38 were susceptible (ITs = 3+ to 4). Chi-squared analysis of the phenotyping results did not deviate from the expected segregation ratio of 3:1 for a single dominant gene (*χ*^*2*^ = 3.49, *p* = 0.062).

### The origin of the alien segment carrying *Lr53* and *Yr35*

Previous studies have reported that the linked resistance genes *Lr53* and *Yr35* were introgressed into common wheat from *T. dicoccoides* and mapped to the short arm of chromosome 6B (Dadkhodaie et al. 2011; Marais et al. 2005a). To verify the origin of the *Lr53* and *Yr35* segment, a comparison was made between the SNPs identified in the RNA-seq data of the donor line 98M71 and those in *T. dicoccoides* (Zavitan), *T. durum* (Svevo), and *T. aestivum* (15 hexaploid wheat varieties). To our surprise, we found that the chromosome 6B in 98M71 had a large number of rare polymorphisms (11,953 SNPs from the start of the chromosome to 687.0 Mb; Table S2) that were absent in all tetraploid and hexaploid wheat accessions. This observation suggests that the chromosome segment carrying *Lr53* and *Yr35* may not originate from *T. dicoccoides*.

**Fig. 2:**
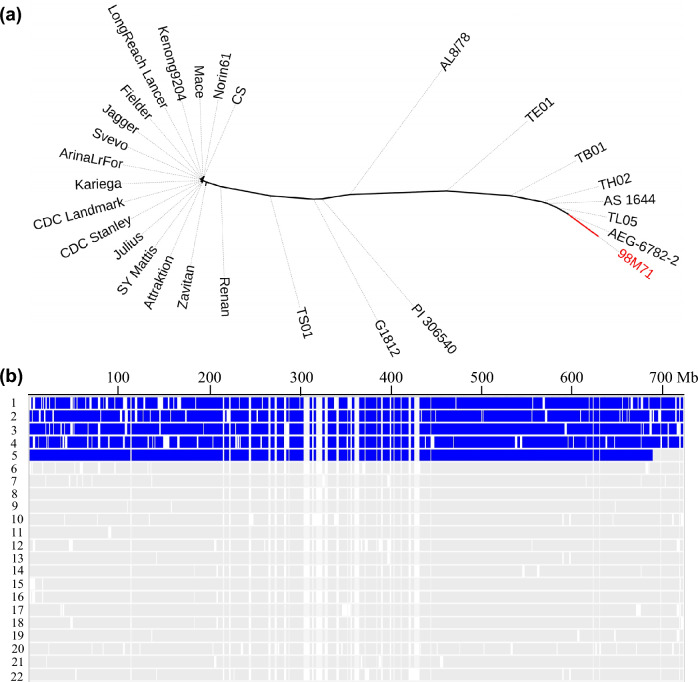
The origin of the alien segment carrying *Lr53* and *Yr35*. **a** Phylogenetic analysis based on 9,294 SNPs (Table S3) identified within the ~ 687.0 Mb segment on chromosome 6B across 11 wheat species. The neighbor-joining method was employed for inferring the evolutionary history, and the resulting tree was visualized using the interactive tree of life (iTOL) v5. The wheat line 98M71 is highlighted in red. **b** Distribution of 8,322 putative *Ae. sharonens/Ae. longissima*-specific SNPs on chromosome 6B. The introgressed segment in 98M71 spans approximately 687.0 Mb, extending from the start of the chromosome to 687.0 Mb. 1, AEG-6782-2; 2, TL05; 3, AS_1644; 4, TH02; 5, 98M71; 6, Zavitan; 7, Svevo; and 8-22, sequenced *T. aestivum* accessions CS, Arina*LrFor*, Attraktion, Fielder, Jagger, Julius, Kariega, Kenong9204, LongReach Lancer, CDC Landmark, Mace, Norin61, Renan, CDC Stanley, and SY Mattis. This figure was generated using the Integrative Genomics Viewer (IGV) software v2.8.9. Vertical lines in blue represent *Ae. sharonens/Ae. longissima*-specific SNPs whereas lines in light gray are normal wheat SNPs. Coordinates are based on CS RefSeq v1.1 (color figure online)

To further explore the potential origin of the *Lr53* and *Yr35* segment, the RNA-seq data of 98M71 were compared with the available genomic sequences of *T. monococcum* (PI 306540), *T. urartu* (G1812), *Ae. sharonensis* (TH02 and AS_1644), *Ae. longissima* (TL05 and AEG-6782-2), *Ae. speltoides* (TS01), *Ae. searsii* (TE01), *Ae. bicornis* (TB01), *Ae. tauschii* (AL8/78), as well as *T. dicoccoides*, *T. durum*, and *T. aestivum*. We focused only on the polymorphisms located within the ~ 687.0 Mb segment on chromosome 6B (based on CS RefSeq v1.1 coordinates) that are polymorphic among the 11 wheat species described above. Based on this approach, a total of 9294 SNPs were identified among the different wheat species (Table S3). A neighbor-joining tree based on these SNPs showed that 98M71 is located in a branch encompassing the accessions of *Ae. longissima* and *Ae. sharonensis* (Fig. 2a), suggesting that the chromosome segment carrying *Lr53* and *Yr35* likely originated from either of these two *Aegilops* species or their derived polyploid species (e.g., *Ae. kotschyi* or *Ae. peregrina*, genome UUSS).

To gain a deeper understanding of the *Lr53* and *Yr35* chromosomal segment, our analysis focused on the specific SNPs that were present in two accessions of *Ae. longissima* (TL05 and AEG-6782-2) and two accessions of *Ae. sharonensis* (TH02 and AS_1644), but absent in other polyploid wheat accessions (*T. dicoccoides*, *T. durum*, and *T. aestivum*). These SNPs are hereafter referred to as *Ae. longissima*/*Ae. sharonensis*-specific SNPs. Through this comparison, we identified 8,322 putative *Ae. longissima*/*Ae. sharonensis*-specific SNPs that were shared with 98M71 on chromosome 6B (Table S4). To visualize the distribution of these SNPs, we plotted these *Ae. longissima*/*Ae. sharonensis*-specific SNPs as blue vertical lines, while other normal wheat SNPs shown in gray (Fig. 2b). This figure revealed that the alien chromosome segment in 98M71 spans a length of approximately 687.02 Mb (from 0 to 687.02 Mb in CS RefSeq v1.1; Fig. 2b). The translocation breakpoint was located between SNPs at positions 687,016,683 bp and 688,295,787 bp (Table S4).

Using the reference genomes of TL05 (*Ae. longissima*) and TH02 (*Ae. sharonensis*), we developed 17 genome-specific primer pairs targeting the 6S/6B genomes across the 687.02-Mb introgressed chromosomal segment (Tables S5 and S6). These primers were used to amplify PCR products from 98M71, which were subsequently sequenced using the Sanger method. The obtained sequences from 98M71 were then subjected to BLASTN searches against the reference genomes of the 11 wheat species mentioned above. All the sequences from 98M71 exhibited greater similarity to *Ae. longissima*/*Ae. sharonensis* than to the other nine wheat species, including *T. dicoccoides* (Table S6). Furthermore, we evaluated ten *Ae. longissima* and *Ae. sharonensis* accessions with five markers derived from the introgressed S segment of 98M71. Most of these accessions exhibited haplotypes identical to those of 98M71 (Table S7). These findings strongly confirm that the chromosome segment carrying *Lr53* and *Yr35* indeed originated from either *Ae. longissima* or *Ae. sharonensis*, or their derived species.

Six PCR markers *pku6B97F* (6.03 Mb; CS RefSeq v1.1), *pku6B1059* (85.79 Mb), *pku6B1851* (209.24 Mb), *pku6B2389* (415.87 Mb), *pku6B2836* (512.19 Mb), and *pku6B613M* (613.11 Mb; Fig. S1) were used to genotype the selected 136 and 117 F_2_ plants from the 98M71 × Avocet-S cross described above. Among the 253 plants evaluated with the six markers, recombination events were observed only between PCR markers *pku6B1851* (209.24 Mb) and *pku6B2389* (415.87 Mb) at a frequency of 1.0% (Tables S8 and S9). These recombination events are probably due to centromeric (Robertsonian) translocations. However, no recombination was detected among the markers *pku6B97F* (6.03 Mb), *pku6B1059* (85.79 Mb), and *pku6B1851* (209.24 Mb) on chromosome arm 6BS in the presence of the *Ph1* gene (Tables S8 and S9). These three markers were completely linked to the phenotypes, confirmed that *Lr53* and *Yr35* were located on chromosome arm 6BS. The translocation breakpoint in 98M71 was defined using PCR markers and pinpointed between markers *pku6B4135* (687.02 Mb) and *pku6B4191* (690.96 Mb; Fig. S1).

We then explored the presence of the alien translocation in PI 682091 (Thatcher-*Lr53*), a Thatcher near-isogenic line carrying *Lr53* and *Yr35* (Marais et al. 2018). Sanger sequencing was performed on seven 6S/6B-genome-specific PCR markers (*pku6B165*, *pku6B97F*, *pku6B756*, *pku6B1059*, *pku6B2836*, *pku6B613M*, and *pku6B4135*; Table S5) across the introgressed segment. The results confirmed that PI 682091 carries the same allele and length of the alien segment as observed in 98M71.

**Fig. 3:**
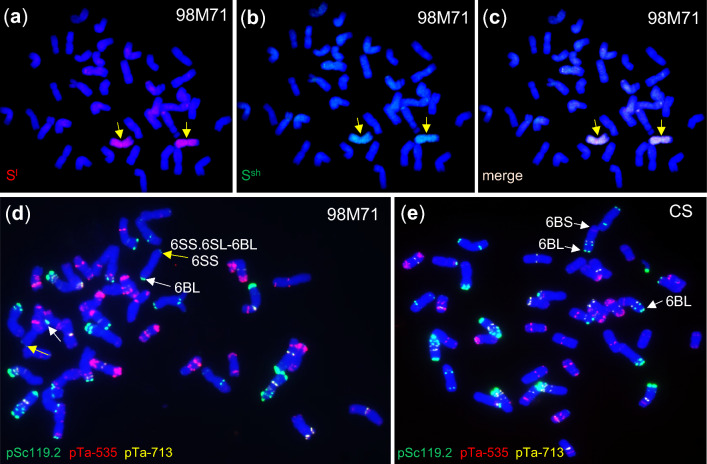
Genomic in situ hybridization (GISH) and fluorescence in situ hybridization (FISH) images of wheat line 98M71. **a**–**c** GISH images of mitotic chromosomes in 98M71. Genomic DNA of *Ae. longissima* (S^l^S^l^) and *Ae. sharonensis* (S^sh^S^sh^) were labeled with the Atto550 NT labeling kit (red) and Atto488 NT labeling kit (green), respectively. Yellow arrows indicate the introgressed 6S chromosomes. **d–e** FISH images of 98M71. Probes pSc119.2 (green), pTa535 (red), and pTa713 (yellow) were used for the hybridization experiments. Genomic DNA of CS was used as blocking DNA. Yellow arrows indicate the Oligo-pSc119.2 signals in the telomeric region of the alien chromosome arm 6SS, and white arrows represent the Oligo-pSc119.2 signals in the telomeric region of wheat (CS) chromosome arm 6BL. 6SS.6SL-6BL, the recombinant chromosome in 98M71. All experiments were repeated three times independently with consistent results (color figure online)

To validate the findings described above, GISH experiments were conducted. The GISH analyses confirmed the presence of the *Ae. longissimi* or *Ae. sharonensis* translocation in 98M71 on chromosome 6B (Fig. 3a–c and S2). Approximately 95% of the physical length of chromosome 6B was replaced by 6S chromatin, while the small wheat 6BL telomeric end accounted for the remaining 5% observed in the images (Fig. 3a–c). This original recombinant chromosome was hereafter referred to as 6SS.6SL-6BL. Further karyotype analysis using FISH revealed strong Oligo-pSc119.2 signals in the telomeric region of the alien chromosome arm 6SS (marked with yellow arrows) in 98M71 (Fig. 3d). In contrast, this pSc119.2 signal was absent in the telomeric region of chromosome arm 6BS in the recurrent parent CS (Fig. 3e), further confirming the presence of the alien translocation in 98M71. Another Oligo-pSc119.2 signal was detected in the telomeric region of chromosome arm 6BL in both 98M71 and CS, indicated by white arrows (Fig. 3d, e).

### Mapping of *Lr53* and *Yr35* using *ph1b*-induced homoeologous recombination

**Fig. 4:**
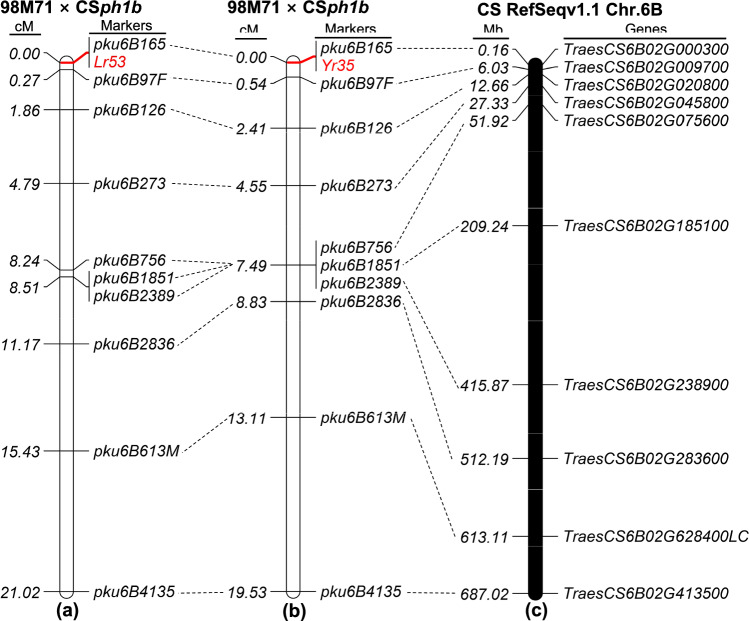
Genetic maps for *Lr53* and *Yr35*. **a** Genetic map for *Lr53*, constructed using 188 F_2_ plants and 10 PCR markers. The values to the left of the PCR markers represent the genetic distances in centimorgans (cM). **b** Genetic map for *Yr35* based on 187 F_2_ plants and 10 PCR markers. The values to the left of the PCR markers indicate the genetic distances in centimorgans (cM). **c** Colinear genomic region on chromosome 6B of Chinese Spring (RefSeq v1.1). The values to the left of the genes are physical locations in megabases (Mb; CS RefSeq v1.1)

To induce recombination between wheat chromosome 6B and the introgressed 6S chromosome segment, a cross was made between the introgression line 98M71 and the CS*ph1b* mutant. The PCR markers *pku6B97F* (6.03 Mb), *pku6B1851* (209.24 Mb), and *pku6B4135* (687.02 Mb) were used to select F_2_ plants that were heterozygous for the introgressed 6S segment. Subsequently, six F_2_ plants were obtained which were homozygous for *ph1b* and heterozygous for the introgressed 6S segment. These plants were self-pollinated to generate F_3_ seeds for genetic mapping. Among the 188 F_3_ plants from the 98M71 × CS*ph1b* cross inoculated with *Pt* race THDB, 132 plants exhibited resistance (ITs = 0; to ;1-) and 56 were susceptible (ITs = 3–4). This distribution corresponded to the segregation ratio of 3:1 expected for a single dominant genetic locus (*χ*^*2*^ = 2.30, *p* = 0.13). All plants were genotyped using ten 6S/6B-genome-specific PCR markers (Table S5) across the introgressed chromosomal segment. By integrating these polymorphic DNA markers with the leaf rust resistance phenotypes, a genetic linkage map for *Lr53* was constructed (Fig. 4a). Based on the linkage results, *Lr53* was mapped to the distal region of chromosome arm 6BS, located 0.27 cM distal to *pku6B97F*, and was completely linked to the marker *pku6B165* (Fig. 4a).

Similarly, seedlings of another 187 F_3_ plants inoculated with *Pst* race CYR34, 130 plants displayed resistance (ITs = 0; to 1), and 57 were susceptible (ITs = 3–4). The observed ratio remained consistent with the expected 3:1 segregation ratio (*χ*^*2*^ = 3.00, *p* = 0.08). These 187 plants were genotyped using the same PCR markers, resulting in the construction of a genetic map for *Yr35* (Fig. 4b). *Yr35* was mapped 0.54 cM distal to *pku6B97F* and was completely linked to *pku6B165*, within the same interval as *Lr53* (Fig. 4a, b).

To determine the localization of *Lr53* and *Yr35* more precisely, we screened an additional 1,571 F_3_ plants with the markers *pku6B97F* (6.03 Mb) and *pku6B165* (0.16 Mb; CS RefSeq v1.1). Within this screening, 15 plants carrying informative recombination events were identified. Based on these recombinants and three additional plants found in the previous screening of 375 plants (188 and 187 individuals), the genetic distance between *pku6B97F* and *pku6B165* was reestimated to be 0.46 cM. The progeny of these 18 plants with informative recombination events were subsequently inoculated with *Pt* race THDB and *Pst* race CYR34, respectively. Using these recombinants and two newly developed markers within the candidate region [*pku6B3127* (3.13 Mb) and *pku6B5555* (5.56 Mb); Table S5], both *Lr53* and *Yr35* were confined to the same 6.03-Mb interval and completely linked to markers *pku6B165*, *pku6B3127*, and *pku6B5555* (Table S5).

### Candidate genes for *Lr53* and* Yr35* in the colinear regions of sequenced wheat genomes

Comparisons among the published reference genomes of *Ae. sharonensis* (TH02 and AS_1644), *Ae. longissima* (TL05 and AEG-6782-2), *T. dicoccoides* (Zavitan), *T. durum* (Svevo), and *T. aestivum* (five hexaploid wheat varieties) revealed potential structural rearrangements and chromosomal inversions within the *Lr53* and *Yr35* candidate region (Fig. S3). These observed rearrangements and inversions in the candidate region across different wheat genomes provide an explanation for the lack of recombinations detected among markers *pku6B165*, *pku6B3127*, and *pku6B5555*, although a large mapping population consisting of 3,892 gametes was used.

The 6.03-Mb candidate region in CS contains 97 annotated genes with high-confidence (*TraesCS6B02G000100*–*TraesCS6B02G009700*). These genes include four annotated NLR genes and 23 receptor-like protein kinases (Table S10). The corresponding regions in the TH02 (*Ae. sharonensis*) and TL05 (*Ae. longissima*) reference genomes, spanning 3.28 Mb and 3.37 Mb, respectively, also contain multiple NLR genes and receptor-like protein kinases (Table S11 and S12). These genes hold significant relevance for this project as NLRs and protein kinases are the most prevalent gene classes associated with disease resistance in plants.

Transcript levels of the candidate genes were analyzed in *Pt*-inoculated and mock-inoculated 98M71 plants at 6 dpi (Table S13). Out of the 119 annotated genes within the candidate region of the TL05 (*Ae. longissima*) genome, 42 genes were expressed in wheat leaves infected with *Pt* race THDB. These expressed genes included one annotated NLR gene and 12 receptor-like protein kinases (Table S13). Among them, transcript levels of *Ae.longissima.TL05.6S01G0003700.1* were significantly higher in *Pt*-inoculated plants (FDR < 0.05, *p-*value < 0.05, and |log_2_fold change|> 1) compared to mock-inoculated controls (Table S13). Conversely, the *Ae.longissima.TL05.6S01G0003400.1* gene exhibited significantly lower transcript levels in *Pt*-inoculated plants relative to mock-inoculated plants (Table S13).

DEGs between *Pst*-inoculated and mock-inoculated 98M71 plants at 6 dpi were also identified using RNA-seq data (Table S14). Among the candidate genes in the target interval in TL05, we found that 49 genes were expressed in wheat leaves infected with *Pst* race CYR34, which included 16 annotated protein kinases and one NLR gene. A total of eight DEGs were significantly upregulated in *Pst*-inoculated plants relative to mock-inoculated plants (Table S14).

To validate these findings from RNA-seq data, the expression levels of the genes *Ae.longissima.TL05.6S01G0003400.1*, *Ae.longissima.TL05.6S01G0003700.1*, *Ae.longissima.TL05.6S01G0003200.1*, and *Ae.longissima.TL05.6S01G0009000.1* were further determined using qRT-PCR. The results confirmed significant upregulation or downregulation (*p* < 0.05) of these genes after *Pt* or *Pst* inoculation compared to mock inoculation (Fig. S4 and S5), supporting the findings obtained from the RNA-seq analysis. In addition, we detected amino acid changes between 98M71 and TL05 for these four DEGs (Table S15), but it was unknown whether *Lr53* and *Yr35* were present in TL05.

### Validation of the *Lr53*- and* Yr35*-linked markers in uncharacterized tetraploid and hexaploid wheat accessions

To determine the value of the tightly linked markers identified in this study for marker-assisted selection, we evaluated a collection of 87 accessions of *T. turgidum* (*T. dicoccon*, *T. dicoccoides*, and *T. durum*) and 149 of *T. aestivum* with the flanking marker *pku6B97F* and three completely linked markers *pku6B165*, *pku6B3127*, and *pku6B5555*. None of these wheat accessions showed haplotypes identical to those of 98M71 and PI 682091 (Table S16). PCR amplification using the marker *pku6B3127* at an annealing temperature of 56˚C resulted in the generation of an 1118-bp fragment in 73 (83.9%) of *T. turgidum* accessions and 53 (35.6%) of the *T. aestivum* accessions (Table S16). Treatment of the amplified PCR products with the restriction enzyme *Ava*I generated two bands of 418-bp and 700-bp for the introgression lines 98M71 and PI 682091 carrying *Lr53* and *Yr35*, while a single band of 1118-bp was observed for the other tetraploid and hexaploid wheat accessions (Fig. S6). No PCR product was amplified from 14 *T. turgidum* and 96 *T. aestivum* accessions (Table S16), indicating the absence of the introgressed segment in these accessions. These results suggest that the marker *pku6B3127* holds significant value in predicting the presence of *Lr53* and *Yr35* in uncharacterized wheat genotypes.

### Transfer of a small alien chromosome segment carrying *Lr53* and* Yr35* into hexaploid wheat cultivar YM21

**Fig. 5:**
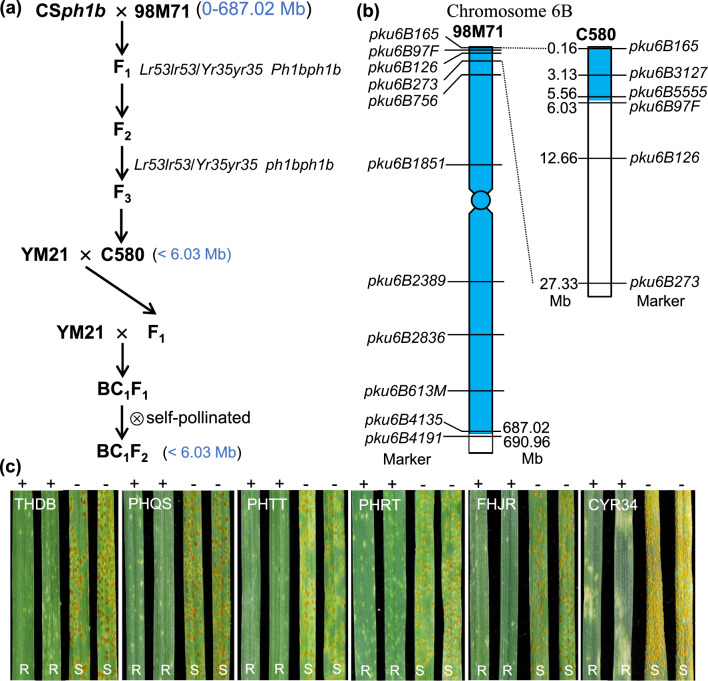
Introgression of the truncated alien segment (< 6.03 Mb) carrying *Lr53* and *Yr35* into the common wheat background. **a** Procedures for generating C580 and transferring the truncated 6S segment into hexaploid wheat. YM21, bread wheat cultivar Yangmai21. **b** Markers were used to estimate the length of the truncated 6S chromosomal segment in C580. The crossover breakpoint in C580 was between markers *pku5555* (5.56 Mb) and *pku6B97F* (6.03 Mb; CS RefSeq v1.1). Blue regions indicate the introgressed alien chromatin, while white rectangles represent *T. aestivum* 6B chromatin. **c** Infection types observed in the new introgression line with the truncated 6S chromosome segment (< 6.03 Mb), as well as the control YM21 and BC_1_F_2_ plants lacking the truncated alien segment. Plants were challenged with five *Pt* races (THDB, PHQS, PHTT, PHRT, and FHJR) and one *Pst* race (CYR34). + indicates the presence of the resistant allele, while − denotes the absence of the resistant allele. R, resistant; S, susceptible

Using *ph1b*-induced homoeologous recombination, we successfully obtained a resistant recombinant line named C580 from the 98M71 × CS*ph1b* mapping population. The procedures for generating C580 and transferring the truncated 6S segment into hexaploid wheat are illustrated in Fig. 5a. In C580, PCR markers *pku6B165* (0.16 Mb), *pku6B3127* (3.13 Mb), and *pku6B5555* (5.56 Mb) showed the 6S (98M71) alleles, whereas markers *pku6B97F* (6.03 Mb), *pku6B126* (12.66 Mb), and *pku6B273* (27.33 Mb) exhibited the *T. aestivum* (CS) alleles (Fig. 5b), indicating the crossover breakpoint occurred between markers *pku6B5555* and *pku6B97F*. Therefore, the size of the truncated 6S chromosome segment in C580 is between 5.56 and 6.03 Mb based on Chinese Spring RefSeq v1.1 coordinates (Fig. 5b). FISH-based karyotype analysis revealed strong Oligo-pSc119.2 signals in the telomeric regions of both chromosome arms 6SS and 6BL in C580 (Fig. S7). The progeny of C580 homozygous for the presence of the truncated alien segment showed high resistance to *Pt* and *Pst* races THDB, PHQS, PHTT, PHRT, FHJR, and CYR34 (similar to 98M71; Fig. S8), whereas plants homozygous for the absence of the alien segment displayed susceptible reactions.

Recombinant C580 with the truncated 6S chromosome segment was crossed and backcrossed once with the susceptible Chinese bread wheat cultivar YM21 (Fig. 5a). Four DNA markers, *pku6B165*, *pku6B3127*, *pku6B5555*, and *pku6B97F*, were used to verify the presence of the truncated 6S chromosome segment in the selected BC_1_F_2_ plants (Fig. S9). The BC_1_F_2_ plants homozygous for the truncated 6S segment exhibited strong levels of resistance (ITs = 0; to 1) against all *Pt* and *Pst* races tested (races THDB, PHQS, PHTT, PHRT, FHJR, and CYR34; Fig. 5c). In contrast, the control plants YM21 and BC_1_F_2_, which lacked the truncated alien segment, displayed susceptible infection types (ITs = 3–4) when challenged with the same races (Fig. 5c). These results suggest that the truncated alien segment possesses a similar resistance profile to the original introgressed segment in 98M71.

## Discussion

### Introgression of rust resistance genes *Lr53* and *Yr35* from *Aegilops* species to bread wheat

Marais et al. (2005a) reported that the rust resistance genes *Lr53* and *Yr35* were introgressed from *T. dicoccoides* into common wheat. In this study, we found that the introgressed segment carrying *Lr53* and *Yr35* was introduced from *Ae. longissima* or *Ae. sharonensis* or their derived species. Four lines of evidence support this conclusion. First, the analysis of SNPs and sequence comparisons (Tables S2-S4, S6) demonstrated that the introgressed chromosomal segment clustered together with *Ae. longissima* and *Ae. sharonensis*. Second, GISH and FISH analyses confirmed the presence of the *Ae. longissima* or *Ae. sharonensis* chromatin in 98M71 on the recombinant chromosome 6B/6S (Fig. 3a–e). Third, the 98M71 × Avocet-S mapping population showed suppression of recombination within the 687.02-Mb introgressed region (except for a few putative centromeric translocations), consistent with the characteristic behavior observed in alien introgressions. Finally, 98M71 and Thatcher-*Lr53* were found to carry the same length of the alien chromosome segment throughout their breeding history, which agrees with the discovery of *Ae. longissima* or *Ae. sharonensis* introgression. The pedigrees of 98M71 and Thatcher-*Lr53* suggest that the introgressed segment passed through at least 6 and 5 backcrosses, respectively, along with several generations of self-pollination to reach their current states. This extensive breeding history would imply multiple opportunities for recombination during the meiosis. However, no recombination events were detected within the introgressed segment present in both 98M71 and Thatcher-*Lr53* during crossing and backcrossing. We cannot rule out the possibility that the original alien segment carrying *Lr53* and *Yr35* was spontaneously introgressed into *T. dicoccoides* from *Ae. longissima* or *Ae. sharonensis* or their derived species, and then this alien segment in *T. dicoccoides* accession 479 was transferred to hexaploid wheat by Marais et al. (2005a).

Previous study by Dadkhodaie et al. (2011) reported that the *Lr53* and *Yr35* chromosome region showed a segregation distortion favoring the susceptible allele. Similar results were observed in the current study in tests for both stripe rust and leaf rust, as evidenced by an excess of susceptible plants (Tables S8, S9). The presence of the alien introgression likely explains the observed segregation distortion in the chromosomal region carrying *Lr53* and *Yr35*. Although segregation distortion can occur in both introgressed alien segments and segments from the same species, it is more commonly observed in the former (Chen et al. 2021). Various examples of segregation distortion have been reported in relation to alien introgressions carrying disease resistance genes. Notable instances include *Lr19* in *Ag. elongatum* (Prins and Marais 1999), *Lr54* and *Yr37* in *Ae. kotschyi* (Marais et al. 2005b), and *Yr34* and *QYrtb.pau-5A* in *T. monococcum* (Chhuneja et al. 2008; Lan et al. 2017).

Previous studies had indicated that the introgressed alien segment carrying *Lr53* and *Yr35* exhibited normal recombination with chromosome 6BS of the wheat line Avocet-S (Marais et al. 2005a), with an estimated recombination rate of 3% (11 recombinants out of 186 individuals) between these two genes (Dadkhodaie et al. 2011). The mapping results positioned *Lr53* and *Yr35* around 3.0 cM apart, approximately 18.9 cM proximal to the SSR marker *gwm191* and 1.1 cM distal to *cfd1* (Dadkhodaie et al. 2011). Using the sequences of the flanking markers, we successfully determined the location of the marker *cfd1*, which is located at ~ 40.70 Mb on chromosome arm 6BS in CS RefSeq v1.1, but we were unable to determine the location of the marker *gwm191* on chromosome arm 6BS (*gwm191* was found on chromosomes 3D and 7A). This mapping region (distal to *cfd1*: 40.70 Mb) appears to encompass our purposed candidate region for *Lr53* and *Yr35* (0–6.03 Mb; CS RefSeq v1.1). However, in the presence of the *Ph1* gene, we did not observe recombination between the wheat chromosome arm 6BS and the introgressed 6S segment, as indicated by the lack of recombination events among markers *pku6B97F*-6.03 Mb, *pku6B1059*-85.79 Mb, and *pku6B1851*-209.24 Mb (Tables S8, S9). This absence of recombination is consistent with the typical behavior observed in alien introgressions, although it can also be caused by inverted chromosome segments. In addition, our investigation demonstrated complete linkage between *Lr53* and *Yr35* in a large mapping population (3,892 gametes) derived from the cross between 98M71 and CS*ph1b* (using *ph1b*-induced homeologous recombination). These contradictory results might be attributed to differences in the susceptible parental lines used. Marais et al. (2005b) reported that the introgressed segment did not pair with chromosome arm 6BS of CS during meiosis. It is possible that the wheat line “Avocet-S” used by Dadkhodaie et al. (2011) carries any gene(s) that promote homoeologous pairing in the presence of *Ph1*, which could explain the observed recombination events in their study. Another possibility is the existence of additional *Lr*/*Yr* genes within the introgression segment that contribute to resistance against different races of *Pt* and *Pst* from various regions. An example of this scenario is the case of the original *Sr32* introgression, where another *Sr* gene (*SrAes1t*) was later discovered to contribute to resistance (Mago et al. 2013). However, this probability is low since the small alien segment (< 6.03 Mb) generated in this study showed the same resistance profile and similar seedling resistance responses as the original introgression (687.02 Mb) in 98M71.

### Genetic mapping of *Lr53* and *Yr35* and identification of their candidate genes

Using our genetic maps and the available reference genomes of hexaploid wheat (CS), *Ae. sharonensis* (TH02), and *Ae. longissima* (TL05), we delimited the region of the *Lr53* and *Yr35* candidate genes to a 6.03-Mb region in CS, a 3.28-Mb region in TH02, and a 3.37-Mb region in TL05, respectively (Tables S10–S12). Comparisons among the reference genomes revealed the presence of chromosomal rearrangements and inversions within the *Lr53* and *Yr35* candidate region, as illustrated in Fig. S3. These structural variations likely account for the observed suppression of recombination among markers *pku6B165*, *pku6B3127*, and *pku6B5555* in the presence of the *ph1b* mutation. However, it is important to consider the possibility that these rearrangements and inversions may also arise from potential assembly errors in the reference genomes for this specific region.

The NLR candidate genes identified within the colinear regions of the CS, TH02, and TL05 reference genomes exhibit both copy number and structural variations (Tables S10–S12). Similar to the *Lr53* and *Yr35* candidate region, deletions, duplications, and rearrangements of NLR genes were reported for previously cloned rust resistance genes in wheat, such as *Lr47* (Li et al. 2023), *Sr13* (Zhang et al. 2017), *Sr21* (Chen et al. 2018), and *SrKN* (Li et al. 2021). As many cloned disease resistance genes in wheat and other plant species encode intracellular NLR proteins (Ellis et al. 2000; Marone et al. 2013; Zhang et al. 2017), the NLR genes within the target region are considered strong candidates for *Lr53* and *Yr35*.

In addition to the NLR candidates, numerous receptor-like protein kinases (Tables S10–S12) were detected in the *Lr53* and *Yr35* candidate region. Protein kinases represent promising candidate genes because they are involved in rust resistance in wheat and its wild relatives, such as *Yr15* (Klymiuk et al. 2018), *Yr36* (Fu et al. 2009), *Sr60* (Chen et al. 2020), and *Lr9* (Wang et al. 2023b). We have prioritized these NLR genes and protein kinases for further functional characterization.

### Relationship between *Lr53* and *Yr35* and other *Lr/Yr* genes on chromosome 6S of *Aegilops* species

Several leaf rust and stripe rust resistance genes were identified on chromosome 6S of *Aegilops* species and transferred to the group 6 chromosomes of common wheat, including *Lr9* (Wang et al. 2023b), *Lr/Yr548* (Sharon et al. 2023), *Lr59* (Marais et al. 2008), linked genes *Lr62* and *Yr42* (Marais et al. 2009), and linked genes *Yr38* and *Lr56* (Marais et al. 2010a). *Lr9*, introgressed into bread wheat from *Ae. umbellulata* (genome UU), is located on chromosome arm 6BL (Wang et al. 2023b), indicating its distinction from *Lr53* and *Yr35*. The rust resistance gene *Lr*/*Yr548*, which was identified in *Ae. sharonensis* and *Ae. longissima*, confers resistance against both *Pt* and *Pst* pathogens (Sharon et al. 2023). *Lr*/*Yr548* was located at position 51.76 Mb on chromosome 6S (*Ae. longissima* AEG-6782-2 reference), which differs from the location of *Lr53* and *Yr35* (0–7.31 Mb; AEG-6782-2 v1.0 coordinates). The absence of *Lr*/*Yr548* in the introgression line 98M71 was also confirmed by using a published diagnostic PCR marker for *Lr*/*Yr548* (Sharon et al. 2023) and BLASTN searches in the 98M71 transcriptome database.

*Lr59*, a leaf rust resistance gene introduced from *Ae. peregrina* (genome UUSS), was initially mapped to chromosome arm 1AL (Marais et al. 2008). Subsequent studies by Pirseyedi et al. (2015) revealed that the distal end of the original introgression differed structurally from chromosome 1AL and was homoeologous to the telomeric region of chromosome 6BS (Pirseyedi et al. 2015). Linked resistance genes *Lr62* and *Yr42* were introgressed from tetraploid *Ae. neglecta* (genome UUMM), with *Lr62* being mapped to the distal ends of chromosome arms 6AS and 6BS (Marais et al. 2018). Linked genes *Yr38* and *Lr56* were transferred from *Ae. sharonensis* into chromosome 6A of bread wheat (Marais et al. 2006). During the process of reducing the length of the introgressed segment, *Lr56* was separated from *Yr38* and mapped to the telomeric region of chromosome 6AS (Marais et al. 2018). The leaf rust resistance genes *Lr53* (the current study),* Lr56*, *Lr59*, and *Lr62* have similar locations in the telomeric region of either 6AS or 6BS, as well as the similar resistance profiles and seedling resistance responses (Marais et al. 2018). These results suggest a potential homeo-allelic relationship among these loci. However, the absence of shared markers and high-resolution genetic maps for *Lr56*, *Lr59*, and *Lr62* has limited our ability to establish the mapping relationship among these genes. Further analysis is required to determine the relationship between *Yr35* and the other two *Yr* genes (*Yr38* and *Yr42*) on chromosome 6S.

### Utilization of *Lr53* and *Yr35* in agriculture

Using the PCR marker *pku6B3127*, it was shown herein that the introgressed segment carrying *Lr53* and *Yr35* is absent in all tested tetraploid and hexaploid wheat genotypes, except for the introgression lines 98M71 and Thatcher-*Lr53*. This finding indicates that the incorporation of the *Lr53* and *Yr35* segment has the potential to benefit a wide range of commercially cultivated wheat varieties.

*Lr53* confers robust resistance against more than 60 individual or mixed *Pt* races from China (the current study), South Africa (Marais et al. 2005a), North America (Marais et al. 2018), India (Raghunandan et al. 2022), and Australia (Dadkhodaie et al. 2011), indicating broad-spectrum resistance to different *Pt* races. The strong and broad-spectrum resistance makes *Lr53* a valuable genetic resource in wheat breeding. On the other hand, while *Yr35* showed effectiveness against 11 *Pst* races from different regions (Dong et al. 2017; Marais et al. 2018), our study identified one stripe rust race virulent on *Yr35*. This indicates limited potential for utilizing *Yr35* in regions where the CYR32 race is prevalent. Therefore, a combination of *Yr35* with other CYR32-effective resistance genes, such as *Yr36* (Fu et al. 2009) and *Yr15* (Klymiuk et al. 2018), is a preferred strategy for breeding wheat cultivars with good resistance.

The newly developed introgression line, which contains a very small alien chromosome segment (< 6.03 Mb), provides similar levels of resistance as the initial introgression line 98M71 (Fig. 5c) and minimizes potential linkage drag. This truncated introgression segment with *Lr53* and *Yr35* presents significant interest for breeding programs, as it confers resistance to two distinct wheat pathogens. However, further studies will be needed to test if *Lr53* and *Yr35* are effective in different bread wheat backgrounds and to test potential pleiotropic effects. If necessary, PCR markers within the candidate region can be used to develop new introgression lines with even smaller alien segments carrying the *Lr53* and *Yr35* genes.

In summary, our study successfully demonstrated that the chromosomal segment carrying *Lr53* and *Yr35* were introgressed into bread wheat from *Ae. longissima* or *Ae. sharonensis* or their S-genome containing species. The development of the newly introgressed line and the closely linked PCR markers will facilitate map-based cloning of these two rust resistance genes and accelerate their deployment in wheat breeding programs.

### Supplementary Information

Below is the link to the electronic supplementary material.

Supplementary file1 (PDF 5040 KB)

Supplementary file2 (XLSX 2847 KB)

## Data Availability

The raw sequencing data reported in this study are archived at the National Genomics Data Center under BioProject accession number PRJCA022411. Data supporting the findings of this study are within the manuscript or the supplementary file.
