# Supplementary material for: Mapping and characterization of rust resistance genes Lr53 and Yr35 introgressed from Aegilops species
Source: Theor Appl Genet. 2024 Apr 28;137(5):113. doi: 10.1007/s00122-024-04616-x (PMC11056342; doi:10.1007/s00122-024-04616-x)
Supplement: Supplementary file 1 — Supplementary file1 (PDF 5040 KB) [file 122_2024_4616_MOESM1_ESM.pdf]

Supplementary Figures

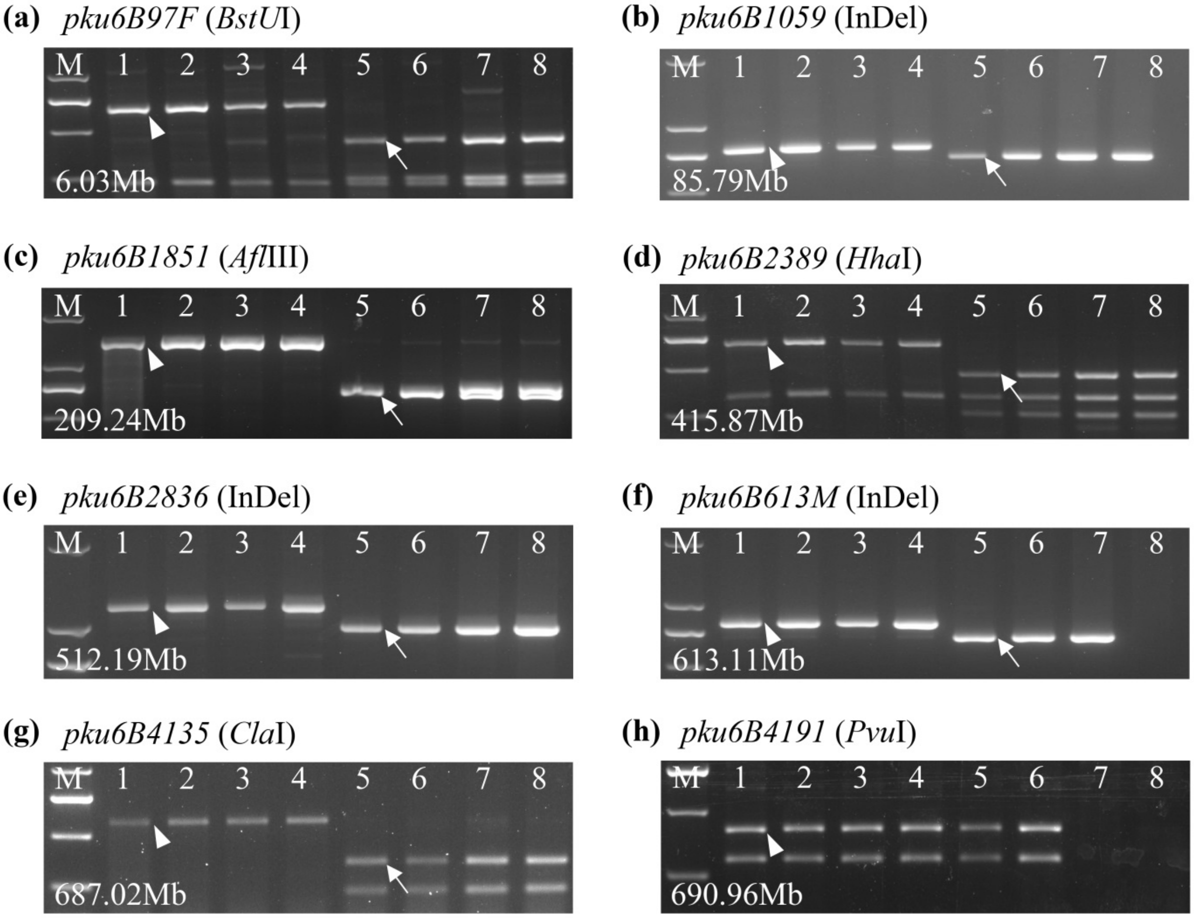

Fig. S1 CAPS markers and InDel markers used to characterize the alien segment present in 98M71. **(a)** CAPS marker *pku6B97F* (6.03 Mb, *BstU*I); **(b)** InDel marker *pku6B1059* (85.79 Mb); **(c)** CAPS marker *pku6B1851* (209.24 Mb, *Afl*III); **(d)** CAPS marker *pku6B2389* (415.87 Mb, *Hha*I); **(e)** InDel marker *pku6B2836* (512.19 Mb); **(f)** InDel marker *pku6B613M* (613.11 Mb); **(g)** CAPS marker *pku6B4135* (687.02Mb, *Cla*I); and **(h)** CAPS *pku6B4191* (690.96 Mb, *Pvu*I). The marker *pku6B4191* was located outside the introgressed segment and was developed based on the *T. aestivum* allele. 1, CS; 2, Avocet-S; 3, Kronos; 4, Zavitan; 5, 98M71; 6, Thatcher-*Lr53*; 7, TH02 (*Ae. sharonens*); 8, TL05 (*Ae. longissima*). M, markers. Coordinates are based on CS RefSeq v1.1. Arrowheads represent wheat bands and arrows represent *Ae. sharonens*/*Ae. longissima* bands.

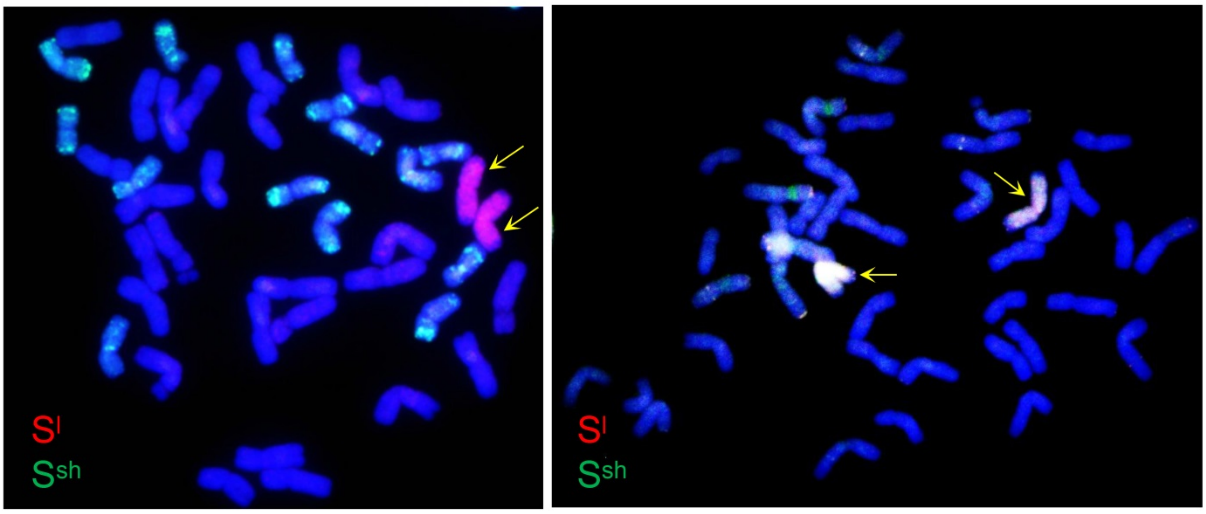

Fig. S2 GISH images of the wheat line 98M71. Genomic DNA of *Ae. longissima* (S^lS^l) and *Ae. sharonensis* ($S^{sh}S^{sh}$) were labelled by the Atto550 NT labelling kit (red) and Atto488 NT labelling kit (green), respectively. Yellow arrows indicate the introgressed 6S chromosomes.

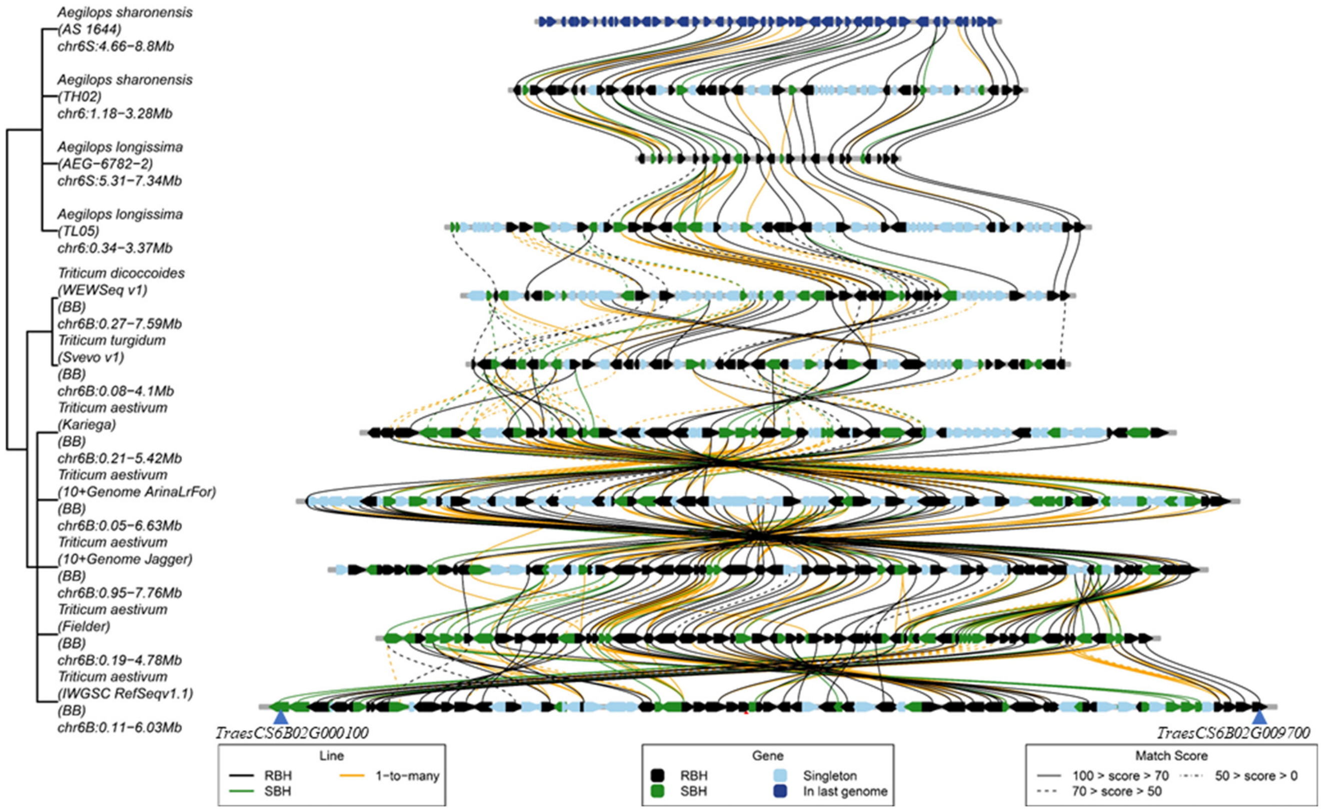

Fig. S3 Micro-collinearity analysis of the *Lr53 and Yr35* candidate region among different *Triticeae* genomes. Candidate genes for our target region were identified from the genome sequences of *Ae. sharonensis* (TH02 and AS_1644), *Ae. longissima* (TL05 and AEG-6782-2), *T. dicoccoides* (Zavitan, WEW), *T. durum* (Svevo), and *T. aestivum* (Kariega, Arina*LrFor*, Jagger, Fielder, CS = IWGSC). Genes are represented by pentagons. Lines indicate similarity among genes. The figure was generated using the Triticeae-GeneTribe database (http://wheat.cau.edu.cn/TGT/) (Chen et al. 2020).

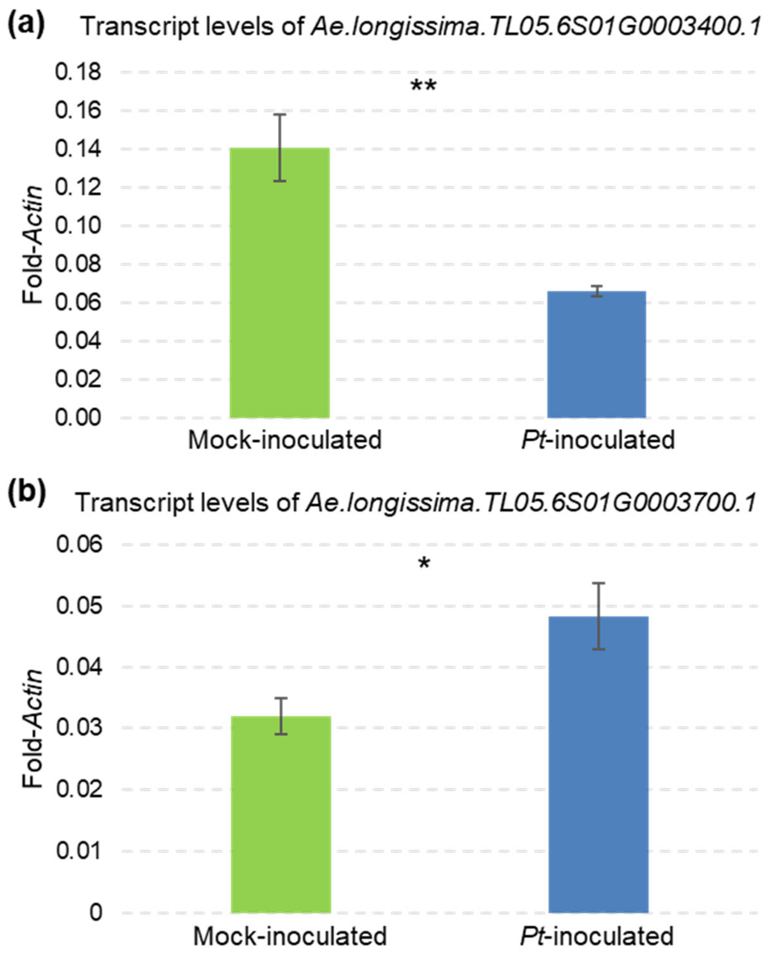

Fig. S4 Transcript levels of **(a)** *Ae.longissima.TL05.6S01G0003400.1* and **(b)** *Ae.longissima.TL05.6S01G0003400.1* in mock-inoculated (green) and *Pt*-inoculated (blue) plants of 98M71 at 6 d post-inoculation (dpi). Seedlings of 98M71 was inoculated with *Pt* race THDB and mock inoculated in two independent growth chambers and leaf samples from different plants were collected at 6 dpi. Transcript levels were expressed as fold-*Actin* (n = 4). Error bars represent standard errors of the mean. *$P < 0.05$; **$P < 0.01$.

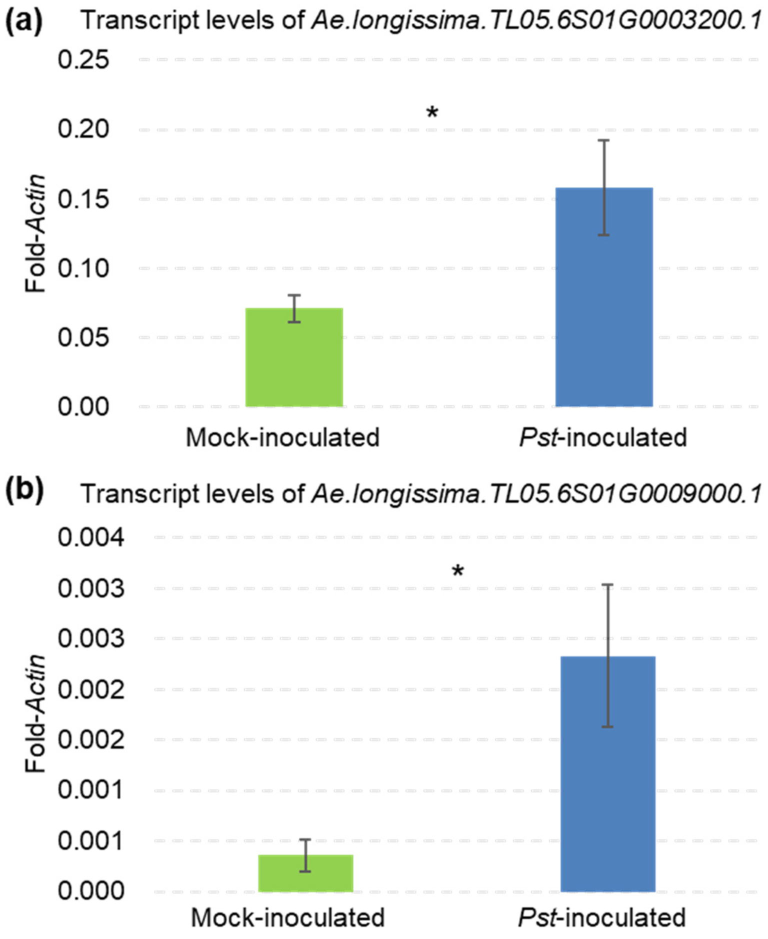

Fig. S5 Transcript levels of **(a)** *Ae.longissima.TL05.6S01G0003200.1* and **(b)** *Ae.longissima.TL05.6S01G0009000.1* in mock-inoculated (green) and *Pst*-inoculated (blue) plants of 98M71 at 6 dpi. Seedlings of 98M71 was inoculated with *Pst* race CYR34 and mock inoculated in two independent growth chambers and leaf samples from different plants were collected at 6 dpi. Transcript levels were expressed as fold-*Actin* (n = 4). Error bars represent standard errors of the mean. *$P < 0.05$.

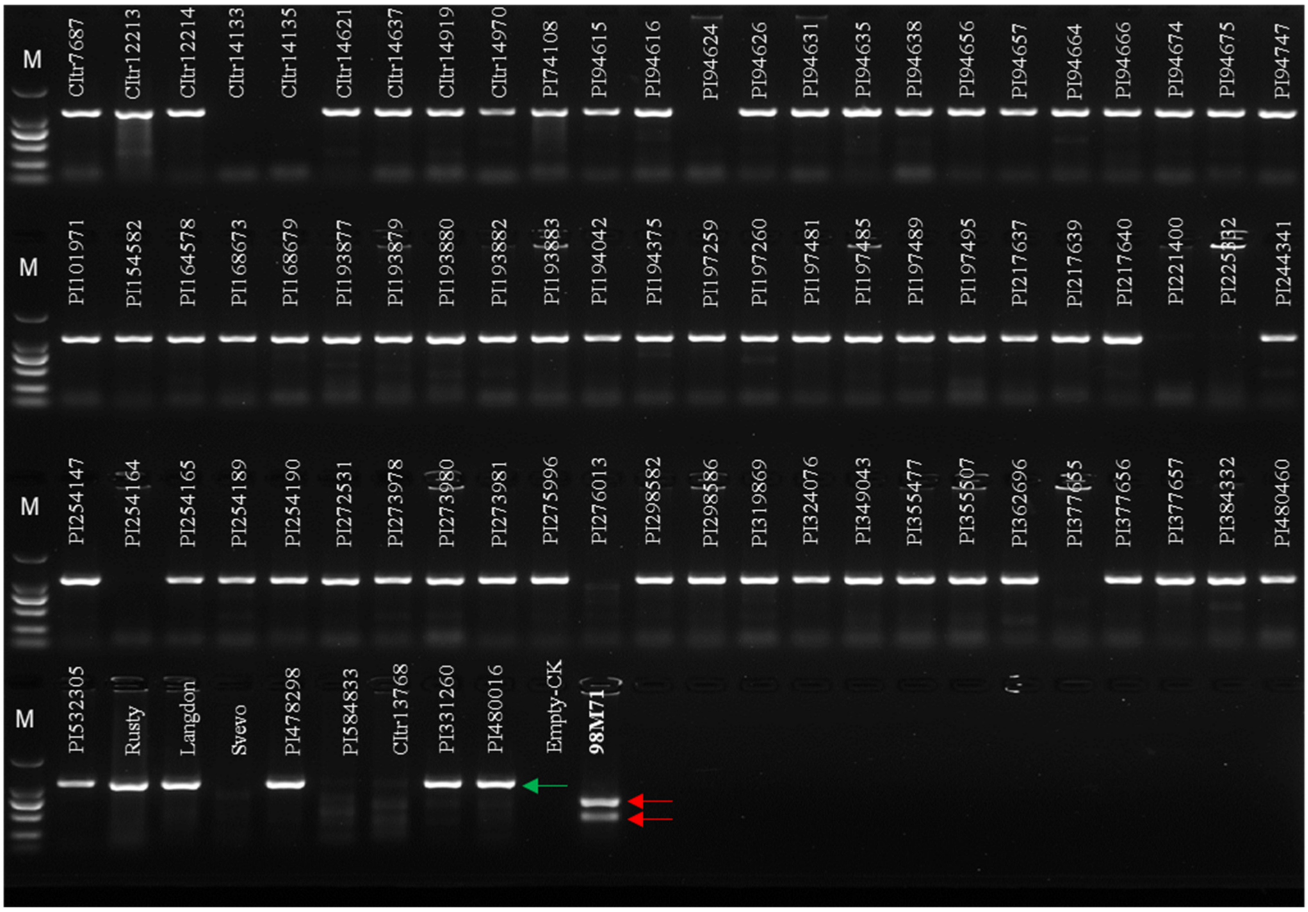

Fig. S6 PCR amplification with the marker *pku6B3127* and treatment of the amplified PCR products with restriction enzyme *Ava*I. The bands of 418-bp and 700-bp (red arrow) are present only in the introgression line 98M71 (Table S16). No amplification product was found in 11 tested *T. turgidum* (e.g. PI 221400, PI225332, and PI 254164). Treatment of the amplified PCR products with restriction enzyme *Ava*I generated a single 1118-bp (green arrow) band for the other tetraploid wheat accessions (e.g. CItr7687, CItr12213, and CItr12214). M, DNA ladders.

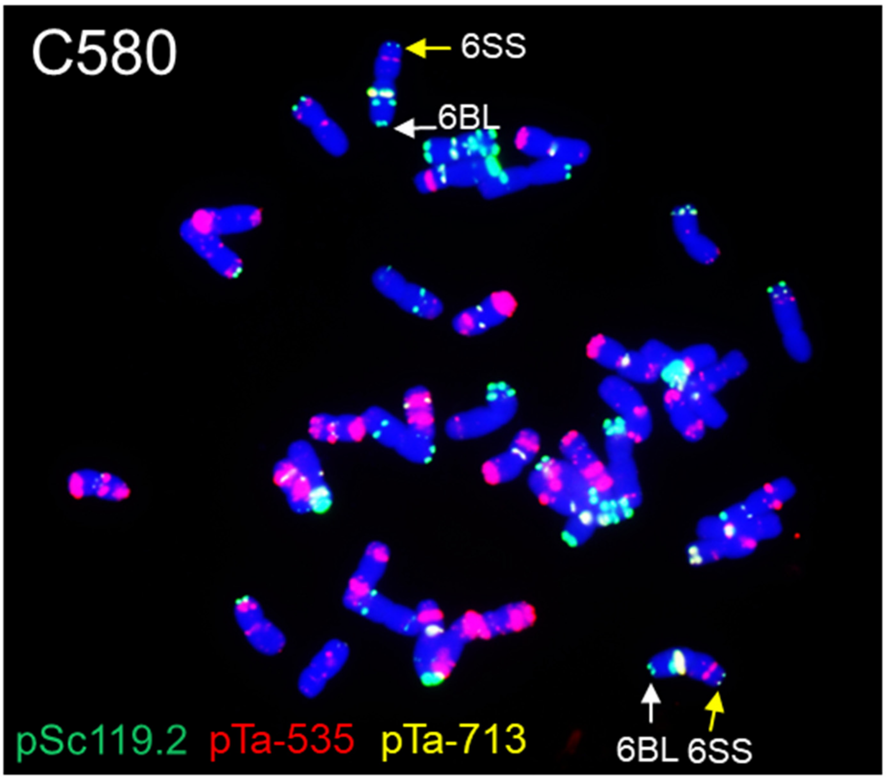

Fig. S7 Fluorescence in situ hybridization (FISH) image of the resistant recombinant line C580. Probes pSc119.2 (green), pTa535 (red), and pTa713 (yellow) were used for the hybridization experiments. Yellow arrows indicate the Oligo-pSc119.2 signals in the telomeric region of the alien chromosome arm 6SS and white arrows represent the Oligo-pSc119.2 signals in the telomeric region of wheat (CS) chromosome arm 6BL.

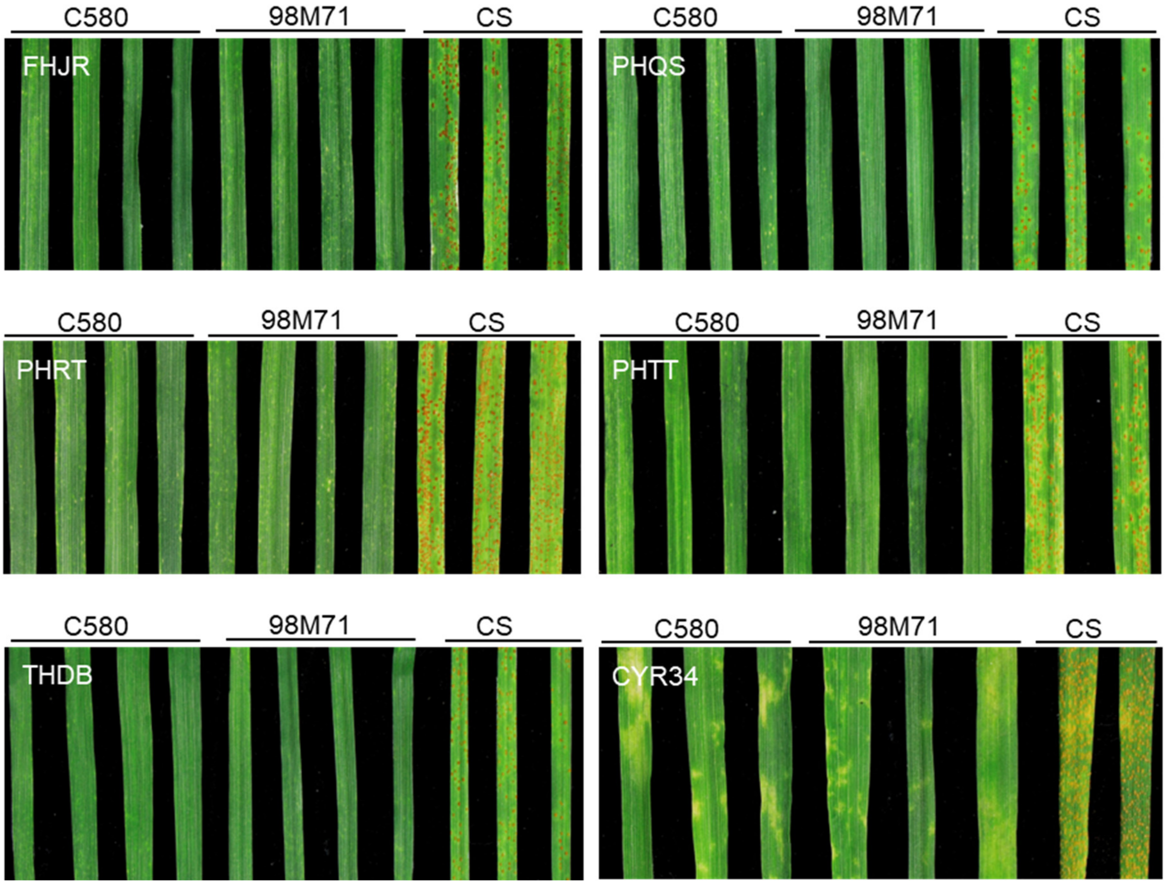

Fig. S8 Infection types observed in the recombinant line C580 (progeny homozygous for the presence of the truncated alien segment), the original introgression line 98M71, and the recurrent parent CS (Chinese Spring). Plants were challenged with five *Pt* races (FHJR, PHQS, PHRT, PHTT, and THDB) and one *Pst* race (CYR34).

(a) *pku6B165* (*Hinc*II) 0.16 Mb

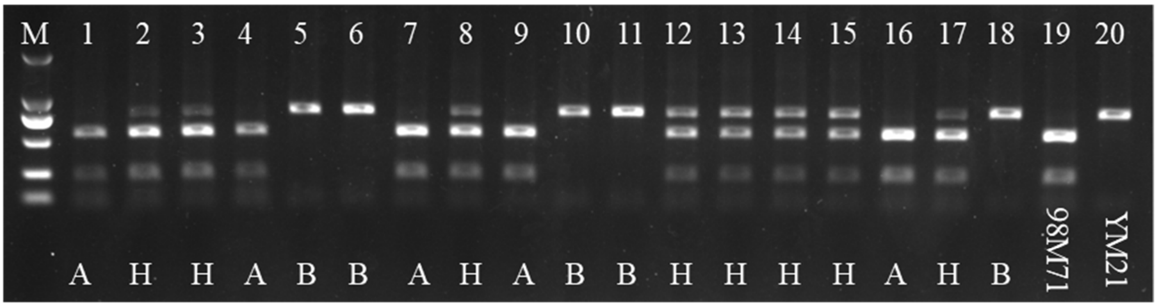

(b) *pku6B3127* (*Ava*I) 3.12 Mb

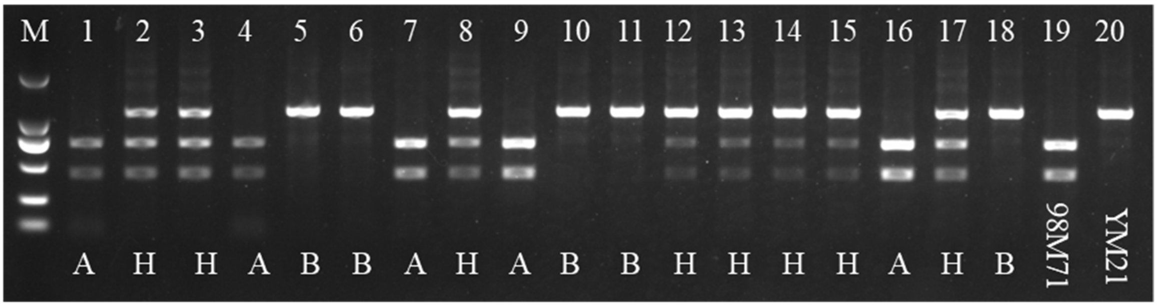

(c) *pku6B5555* (*Pvu*II) 5.56 Mb

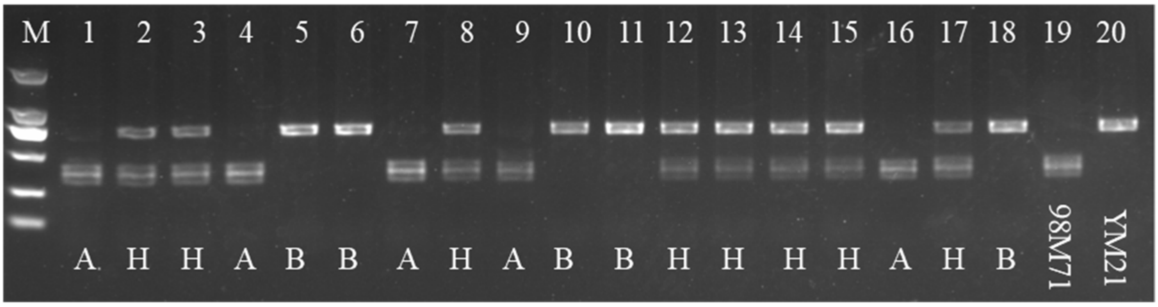

(d) *pku6B97F* (*BstU*I) 6.03 Mb

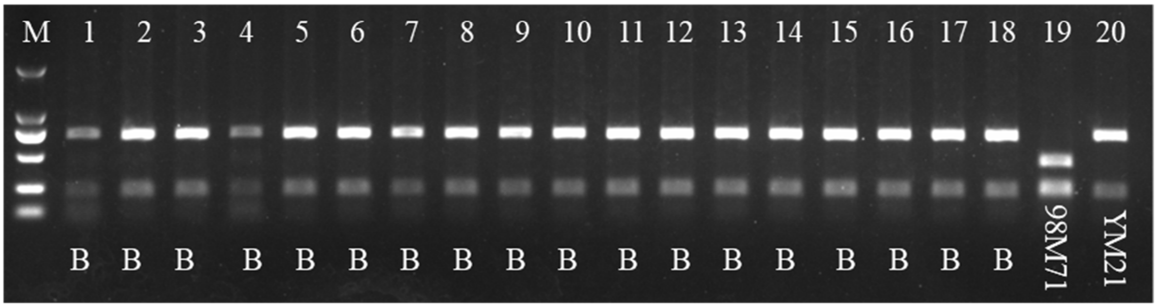

Fig. S9 Markers used to confirm the presence or absence of the truncated 6S in BC_1F_2 plants. **(a)** CAPS marker *pku6B165* (0.16 Mb, *Hinc*II); **(b)** CAPS marker *pku6B3127* (3.12 Mb, *Ava*I); **(c)** CAPS marker *pku6B5555* (5.56 Mb, *Pvu*II); and **(d)** CAPS marker *pku6B97F* (6.03 Mb, *BstU*I). 1-18, BC_1F_2 plants from the YM21 × C580 cross; 19, 98M71; 20, YM21; M, markers. A, resistant (98M71) allele; B, susceptible (YM21) allele; H, heterozygous. Coordinates are based on CS RefSeq v1.1.

References

Chen Y, Song W, Xie X, Wang Z, Guan P, Peng H, Jiao Y, Ni Z, Sun Q, Guo W (2020) A collinearity-incorporating homology inference strategy for connecting emerging assemblies in the Triticeae tribe as a pilot practice in the plant pangenomic era. Molecular Plant 13:1694-1708
